# Single‐Subject Network Analysis of FDOPA PET in Parkinson's Disease and Psychosis Spectrum

**DOI:** 10.1002/hbm.70253

**Published:** 2025-06-12

**Authors:** Mario Severino, Julia J. Schubert, Giovanna Nordio, Alessio Giacomel, Rubaida Easmin, Nick P. Lao‐Kaim, Pierluigi Selvaggi, Zhilei Xu, Joana B. Pereira, Sameer Jauhar, Paola Piccini, Oliver Howes, Federico Turkheimer, Mattia Veronese, FDOPA PET Imaging Working Group Consortium, Ilinca Angelescu, Micheal Bloomfield, Ilaria Bonoldi, Faith Borgan, Tarik Dahoun, D’Ambrosio Enrico, Arsime Demjaha, Jecek Donocik, Alice Egerton, Stephen Kaar, Euitae Kim, Seoyoung Kim, James Maccabe, Julian Matthews, Robert McCutcheon, Philip McGuire, Chiara Nosarti, Matthew M. Nour, Maria Rogdaki, Grazia Rutigliano, Ekaterina Shatalina, Peter S Talbot, Luke Vano

**Affiliations:** ^1^ Department of Information Engineering University of Padua Padova Italy; ^2^ Department of Neuroimaging, Institute of Psychiatry Psychology & Neuroscience, King's College London London UK; ^3^ Centre for Neurodegeneration and Neuroinflammation, Division of Brain Sciences Imperial College London London UK; ^4^ Department of Translational Biomedicine and Neuroscience University of Bari Aldo Moro Bari Italy; ^5^ Division of Neuro, Department of Clinical Neuroscience Karolinska Institute Stockholm Sweden; ^6^ Department of Psychosis Studies, Institute of Psychiatry Psychology & Neuroscience, King's College London London UK; ^7^ Psychiatric Imaging Group, MRC London Institute of Medical Sciences, Hammersmith Hospital Imperial College London London UK; ^8^ South London and Maudsley NHS Foundation Trust London UK

**Keywords:** FDOPA, molecular connectivity, network analysis, Parkinson's disease, PET, schizophrenia

## Abstract

Greater understanding of individual biological differences is essential for developing more targeted treatment approaches to complex brain disorders. Traditional analysis methods in molecular imaging studies have primarily focused on quantifying tracer binding in specific brain regions, often neglecting inter‐regional functional relationships. In this study, we propose a statistical framework that combines molecular imaging data with perturbation covariance analysis to construct single‐subject networks and investigate individual patterns of molecular alterations. This framework was tested on [18F]‐DOPA PET imaging as a marker of the brain dopamine system in patients with Parkinson's Disease (PD) and schizophrenia to evaluate its ability to classify patients and characterize their disease severity. Our results show that single‐subject networks effectively capture molecular alterations, differentiate individuals with heterogeneous conditions, and account for within‐group variability. Moreover, the approach successfully distinguishes between preclinical and clinical stages of psychosis and identifies the corresponding molecular connectivity changes in response to antipsychotic medications. Mapping molecular imaging networks presents a new and powerful method for characterizing individualized disease trajectories as well as for evaluating treatment effectiveness in future research.

## Graphical abstract

We propose a statistical framework combining molecular imaging and perturbation covariance analysis to build single‐subject networks, identifying molecular alterations in brain disorders. This method tests whether it can characterize altered molecular patterns and distinguish disorders through classification.

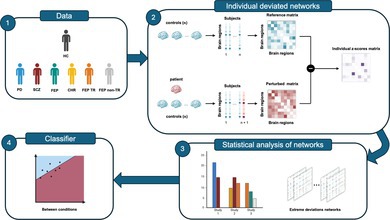

## 1 Introduction

Positron emission tomography (PET) combined with the radiolabelled tracer 6‐[18F]fluoro‐L‐dopa (hereafter, FDOPA) has been extensively utilized to image the dopaminergic system in vivo in the brain (Youdim et al. 2006). The accumulation of FDOPA in the brain parenchyma reflects the tracer's transport, decarboxylation into radiolabelled dopamine, and subsequent vesicular uptake in the nigrostriatal presynaptic nerve terminals. FDOPA was initially employed to quantify the integrity of nigrostriatal dopamine in subclinical models of neuronal damage and Parkinson's Disease (PD) (Calne et al. 1985). In psychiatry, FDOPA PET has been extensively applied to assess the role of the dopaminergic system in the pathophysiology of various psychiatric conditions, including schizophrenia (*American Journal of Psychiatry* 1991), bipolar disorder (Jauhar et al. 2017), 22q11.2 deletion syndrome (Rogdaki et al. 2023), attention‐deficit/hyperactivity disorder (ADHD) (Ernst et al. 1998), and substance dependence (Bloomfield et al. 2014), providing insights into the dopaminergic mechanisms underlying psychotic and other clinical symptoms. Previous studies have also shown that FDOPA is a promising biomarker for psychosis, with the potential to be translated into clinical practice (Rogeau et al. 2025). Furthermore, studies have linked presynaptic dopaminergic activity measured with FDOPA PET to treatment response (TR) in psychosis, indicating it can differentiate between patients likely and unlikely to respond to first‐line antipsychotic drugs (Veronese et al. 2021).

Traditional statistical analysis methods in FDOPA PET research have primarily focused on quantifying absolute tracer uptake within specific brain regions (e.g., the striatum) or individual voxels (Gunn et al. 2001). While this approach provides valuable insights into localized brain activity by identifying abnormal molecular deposition, it has inherent limitations. One of these limitations is that univariate analysis of regions of interest relies on predefined anatomical or functional regions, potentially overlooking distributed effects across the brain (Gentili et al. 2021). Similarly, voxel‐wise analysis examines each voxel independently, often leading to issues with multiple comparisons and inflated Type I error rates, missing inter‐relationships between regions (Scarpazza et al. 2015). These limitations, together with evidence that brain disorders show large‐scale brain network dysfunctions rather than regional alterations (Seeley et al. 2009), suggest there is a need for complementary methodologies to capture molecular interactions among brain regions.

Network‐based approaches, in particular molecular connectivity, can address these limitations, as it allows simultaneous assessment of variations in the relationships between multiple brain regions to investigate brain connectivity (Fornito and Bullmore 2015). This umbrella term has been used in the literature to encompass the statistical interdependencies between regional measurements derived from molecular imaging techniques (Sala et al. 2023). Moreover, a recent literature review has highlighted how connectivity‐based methods are transforming the study of neurotransmission through molecular imaging, enhancing our understanding of neurotransmission processes and offering deeper insights into brain function and disease mechanisms (Severino et al. 2025).

Molecular connectivity has already been investigated using the FDOPA radiotracer. A study has shown that it exhibits greater specificity in revealing the mesotelencephalic system compared to metabolic connectivity derived from [18F]‐FDG (Verger et al. 2020). Furthermore, FDOPA‐based connectivity has demonstrated high test–retest reproducibility (Veronese et al. 2019). Traditional molecular connectivity analysis utilizing static PET data is limited to constructing a group‐level covariance matrix (Yakushev et al. 2017). In contrast, we propose a statistical framework to construct single‐subject networks for investigating FDOPA inter‐relationships across the whole brain, while taking into consideration individual heterogeneity (Liu et al. 2016), thereby overcoming the need for dynamic PET acquisitions to generate individualized network maps. To validate this method, we applied it to static FDOPA PET data across multiple subject groups with distinct brain disorders. The goal was to determine whether the framework can reveal FDOPA PET network patterns associated with established diagnoses and to assess its effectiveness in distinguishing patients diagnosed with different brain conditions.

The work is structured into three independent studies. Study 1 investigated the differential FDOPA covariance patterns obtained from patients with PD and chronic schizophrenia (SCZ). This analysis aimed to show distinct network deviations associated with these two disorders, which are known to affect the dopaminergic system in opposite directions (de Lau and Breteler 2006; Howes et al. 2024). Study 2 examined deviations from normality for individuals at clinical‐high risk (CHR) for psychosis, experiencing first episode psychosis (FEP), and SCZ. This study sought to identify the differences in network deviations across different stages of psychosis. Lastly, Study 3 evaluated the sensitivity of the proposed framework in detecting differences between individuals with FEP according to their TR to standard antipsychotics (i.e., TR vs. non‐TR). Additionally, we assessed the impact of antipsychotic treatment on network deviation patterns in TR.

## 2 Materials and Methods

### 2.1 FDOPA PET Acquisition and Participants

All the data used in this study consisted of FDOPA PET images in the PET‐NODE data repository available at the Institute of Psychiatry Psychology and Neuroscience (IoPPN) at King's College London (KCL). All imaging sessions were acquired with a continuous dynamic acquisition without blood sampling. Following radiotracer administration, data were acquired for 90–95 min using a Siemens Biograph True Point HI‐REZ6 PET/CT system, with the exception of a dataset that was acquired with a different tomograph, namely an ECAT/EXACT3D: Siemens/CTI PET system. All participants received carbidopa (150 mg) and, except for the patients with PD, entacapone (400 mg) orally 1 h before imaging. Both drugs enhance the signal‐to‐noise ratio (SNR) of the tracer uptake in brain tissue by reducing the peripheral formation of radiolabelled dopamine and its metabolites (Veronese et al. 2021). The FDOPA radiotracer (injected dose ranging from 113.97 to 184.2 MBq) was administered by intravenous bolus injection after acquiring a brain CT or MRI for attenuation correction, depending on the scanner availability at each imaging site. PET data reconstruction varied across imaging sites, but consistently included correction for random noise, scatter and tissue attenuation. The parametric image for each scan was finally normalized into MNI standard coordinates using the participant's PET summation image to calculate the image transformation field (nonlinear transformation). More detailed information regarding PET acquisition and processing methods is available elsewhere (Nordio et al. 2023; Egerton et al. 2010). After processing and quality control, PET images were quantified as the standardized uptake value ratio (SUVr), calculated as the ratio of the tracer activity in 83 regions (as per the Hammersmith atlas (Hammers et al. 2003)) to that in the reference region (i.e., mean cerebellar FDOPA PET activity). The interval from 60 to 75 min post‐injection was used for the SUVr calculations as it has been demonstrated to be a valid proxy of dopamine synthesis capacity and tracer uptake (Veronese et al. 2021; Kumakura and Cumming 2009). The FDOPA quantification was performed in original subject PET space, as defined by the summed image after motion correction, to avoid any alteration that might have been introduced with the spatial normalization. All research protocols for data acquisition were approved by local ethics committees and institutional revision boards. Informed written consent was obtained from all the participants, and the studies were conducted following the Declaration of Helsinki and Good Clinical Practice. A total of 234 FDOPA PET scans from existing studies were used. The dataset included 33 PD patients, 105 participants at the different stages of psychosis (i.e., 31 with SCZ, 25 with FEP, 18 of whom were also assessed at follow‐up and 49 at CHR for psychosis), 71 healthy controls (HCs) and 7 further HCs acquired both at baseline and follow‐up (test–retest). The PD dataset comprised patients with idiopathic PD, sourced from the FP7 EC Transeuro program cohort (http://www.transeuro.org.uk/). All patients satisfied Queen Square Brain Bank criteria for PD diagnosis (Hughes et al. 1992). Motor severity was assessed by 2 experienced raters using the motor sub score of the Unified Parkinson's Disease Rating Scale (UPDRS‐III) (Goetz et al. 2008) and the Hoehn and Yahr scale (Hoehn and Yahr 1967) in the practically defined off‐medicated state. Patients were excluded for dementia (Mini‐Mental State Examination score < 26), atypical or secondary parkinsonism, and standard MRI exclusion criteria such as the presence of metallic implants and pregnancy. Further details regarding the research protocol and subject inclusion criteria are provided in the original reference (Li et al. 2018). The SCZ dataset included patients recruited from KCL. Inclusion criteria mandated that participants be aged between 18 and 65 years, meet the Diagnostic and Statistical Manual of Mental Disorders (DSM‐5) criteria for schizophrenia or schizophreniform disorder, and be capable of understanding and consenting to the study procedures. The Mini International Neuropsychiatric Interview (MINI) (Sheehan 1998) was used to aid clinical diagnosis. Medication history and antipsychotic response were recorded through a structured interview and review of medical records. Additional details on clinical assessments and diagnostic criteria are described in (Egerton et al. 2021). The CHR dataset included participants recruited from two early detection services: OASIS (Outreach and Support in South London), part of the South London and Maudsley NHS Trust, and CAMEO (Cambridge Early Onset Service), affiliated with the Cambridge and Peterborough NHS Trust. Participants were included if they met operationalized criteria for CHR for psychosis, as determined with the comprehensive assessment of at‐risk mental states (CAARMS (Yung et al. 2005)). Additional inclusion criteria required no current/past diagnosis of psychotic/neurological disorder assessed with the structured clinical interview for diagnosis (SCID (Glasofer et al. 2015)); no current substance misuse or dependence meeting Diagnostic and Statistical Manual of Mental Disorders (Fourth Edition) criteria; and no contraindication to MRI or PET scanning. Further details are reported in the original reference (Egerton et al. 2013). The FEP dataset consisted of patients recruited from clinical services catering to individuals presenting with a first episode of psychosis in South and West London. Inclusion criteria were a diagnosis of a psychotic disorder according to International Statistical Classification of Diseases and related health problems version 10 (ICD 10) criteria (*The ICD‐10 Classification of Mental and Behavioural Disorders* 1993) and requiring treatment with antipsychotic medication as determined by the treating clinician. Comprehensive details about the research protocol are provided in the original reference (Jauhar et al. 2019). A subset of these subjects (*n* = 18) was also assessed after a follow‐up period. These subjects had undergone antipsychotic treatment at a therapeutic dose, following the Maudsley Prescribing Guidelines (Taylor et al. 2012) for a minimum of 4 weeks before determining treatment response (Agid et al. 2003). Follow‐up continued for a minimum of 6 months to identify any delayed TR in patients who were initially classified as non‐responders at 4 weeks. Thus, this dataset included both subjects who responded to pharmacological treatment (TR) and who did not (non‐TR). Detailed information regarding the research protocol, subject inclusion criteria, and specifications of the medication treatment and response can be found in the original reference (Jauhar et al. 2019). Finally, the HCs comprised test–retest imaging data that was the only dataset acquired with a different tomograph as explained before. There were no missing data in our dataset, and no data imputation was performed during the analysis.

Demographic and clinical characteristics of all participants from both datasets are summarized in Table 1.

**TABLE 1:** Demographic and clinical characteristics of the participants from all datasets.

Group	HC	HC test retest	PD	SCZ	CHR	FEP baseline	FEP follow‐up
Study	Reference cohort (used in all studies)	Test retest	1	1 and 2	2	2 and 3	3
N. subjects	71	7	33	31	49	25	18
Age mean (SD)	27.0 (4.75)	n.a.	55.1 (7.0)	30.8 (10.93)	22.2 (4.26)	26.0 (4.5)	24.7 (3.29)
Sex (male: female)	38:33	7:0	27:6	25:6	27:22	23:2	14:4
N. TR: non‐TR	/	/	/	/	/	13:12	12:6
Scanner	Hi‐Rez Biograph 6 (PET/CT)	ECAT/EXACT3D PET	Hi‐Rez Biograph 6 (PET/CT)	Hi‐Rez Biograph 6 (PET/CT)	Hi‐Rez Biograph 6 (PET/CT)	Hi‐Rez Biograph 6 (PET/CT)	Hi‐Rez Biograph 6 (PET/CT)

Abbreviations: CHR, clinical high risk; FEP, first episode psychosis; HC, healthy controls; PD, Parkinson's disease; SCZ, chronic schizophrenia; TR, treatment response.

### 2.2 Network Perturbation Approach (Theory)

**FIGURE 1:**
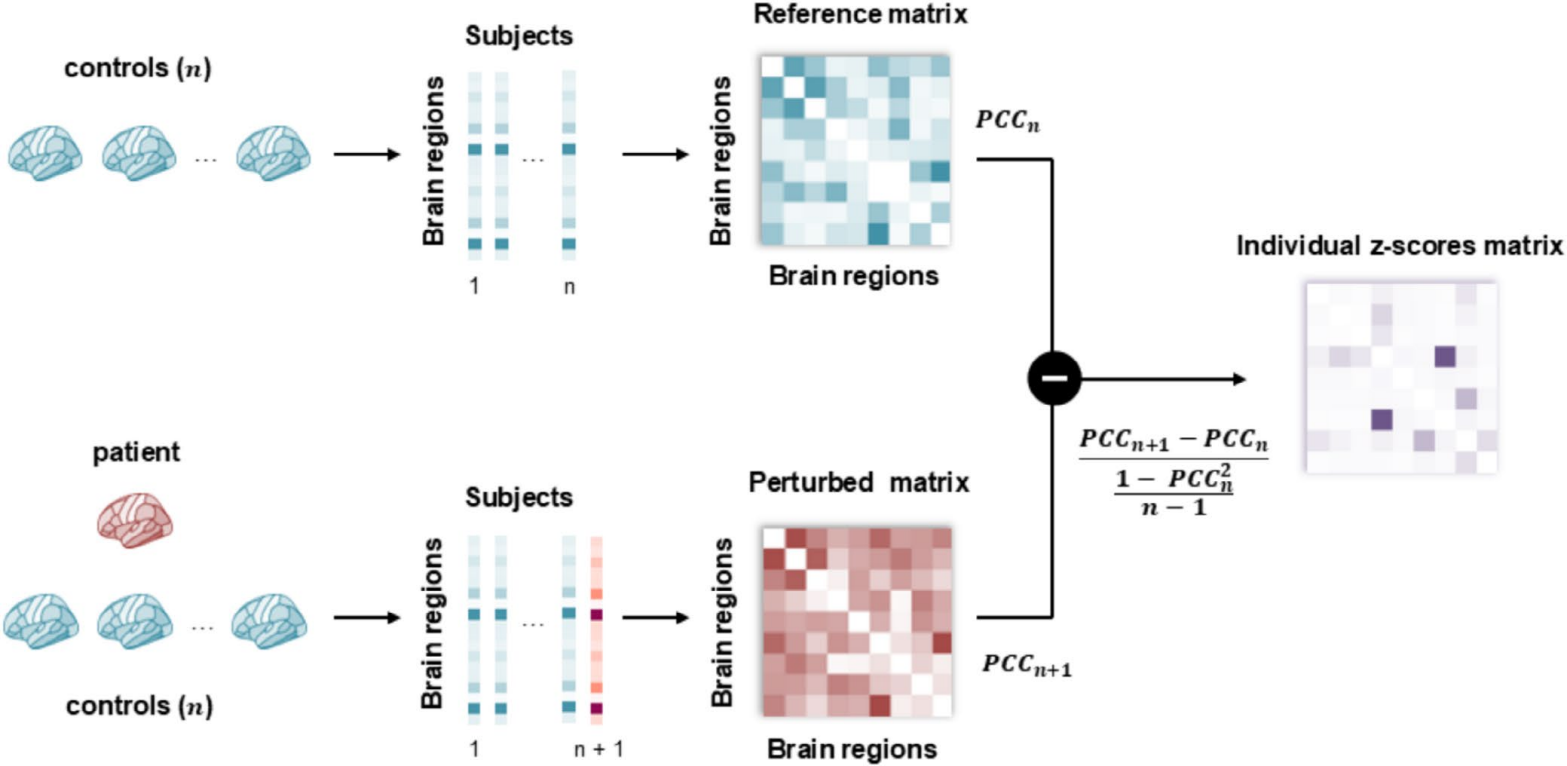
The workflow for constructing individual molecular networks. A reference molecular covariance matrix is first constructed using n healthy controls, with nodes representing brain regions and edges representing the partial correlation coefficients (${\mathrm{PCC}}_{n}$) of the data for each pair of brain regions. A patient subject is then added, and a new covariance network, called the perturbed network (${\mathrm{PCC}}_{n+1}$), is constructed. The individual *z*‐scores matrix of each patient is defined as the *z*‐score of the difference between the perturbed network and the reference network.

To construct individual connectivity networks using static PET data, we adapted a recently developed framework originally designed for single‐sample gene expression networks (Liu et al. 2016) and previously applied to brain structural covariance data (Liu et al. 2021). This approach models individual differences based on normative modeling at the group level. We translated this concept to FDOPA PET imaging, as illustrated in Figure 1.

First, a reference covariance network is constructed using the data from the control group by performing partial Pearson correlation analysis between the data of each region pair, considering possible effects of covariates. In this network, nodes represent the regions, and edges represent the strength of the connections between the nodes, essentially quantified by the Pearson Correlation Coefficient (PCC). Thus, the reference network captures the common characteristics of all subjects included in the reference group. Next, a single subject from the patient group is added to the reference control group, and another network is constructed using partial Pearson correlation. This new network is termed as the perturbed network. The difference between the perturbed network and the reference network, denoted as ${\text{ΔPCC}}_{n}={\mathrm{PCC}}_{n+1}-{\mathrm{PCC}}_{n}$, is calculated to obtain a differential network, which characterizes the specific features of the additional subject against the reference group. If the single subject is similar to the reference cohort, then the perturbation of the PCC on any edge, after adding the subject to the reference cohort, will be insignificant. Conversely, if there are notable differences between the single subject and the reference cohort, adding the subject to the reference cohort will be associated with significant changes in the PCC on edges in the perturbed network. The ΔPCC values of the differential network follow a symmetrical distribution, termed the “volcano distribution”, statistical *Z*‐ or *U*‐tests can be used to evaluate the significance level of each ΔPCC, due to the central limit theorem (Rice 2007). The null hypothesis is that ${\text{ΔPCC}}_{n}$ is equal to the population mean of ${\text{ΔPCC}}_{n}$ and thus we have:

$$Z=\frac{∆{\mathrm{PCC}}_{n}-\mu_{∆\mathrm{PCC}}}{\sigma_{∆\mathrm{PCC}}}=\frac{∆{\mathrm{PCC}}_{n}}{\frac{1-{\mathrm{PCC}}_{n}^{2}}{n-1}}$$

where n denotes the total number of subjects in the reference group, while $\mu_{∆\mathrm{PCC}}$ and $\sigma_{∆\mathrm{PCC}}$ are the mean and standard deviation of the differential network ΔPCC. For a large n, the mean and the standard deviation of ${\text{ΔPCC}}_{n}$ for the population are $\mu_{∆\mathrm{PCC}}=0$ and $\sigma_{∆\mathrm{PCC}}=\frac{1-{\mathrm{PCC}}_{n}^{2}}{n-1}$ (Liu et al. 2016). Thus, the Z matrix essentially represents the level of abnormality in the connectivity, where each edge exhibits a level of variation in molecular activity, leading to deviations from the normal values observed in the control group. The *p*‐value for each edge can be derived directly from the *z*‐scores (Liu et al. 2016).

### 2.3 Networks' Metrics

From the single‐subject matrices, network metrics are extracted to characterize deviation patterns at both the subject and region levels. The individual deviation matrices are thresholded at |*z*| > 4.13, identifying *z*‐scores (edges of the matrix) that are deemed “extreme” relative to the reference control group. The *z*‐score threshold was set to correspond to a Bonferroni‐corrected significance level of 0.05. Subsequently, the following metrics are computed:

- Subject extreme deviations (SED): Let $A_{j}$ denote a symmetric m×m matrix representing deviations for a given subject j, where m are the number of regions. The proportion of extreme deviations for each subject is quantified as $\mathrm{SED}=\frac{T}{C}$ where T is the sum of the absolute value of the thresholded *z*‐scores in the lower triangular portion of the matrix and $C=\frac{m\left(m-1\right)}{2}$ represents the number of elements in the lower triangular portion. This metric quantifies the number of altered connections of each subject.
- Regional extreme deviations (RED): Let $A_{j}$ again represent a symmetric m×m matrix of deviations for a given subject j. For each subject j, we want to compute the mean of the *z*‐scores in each row of matrix $A_{j}$. The mean of the elements in the i *‐th* row of matrix $A_{j}$ for subject j is given by:

$$\mu_{\mathrm{ij}}=\frac{1}{m}\sum_{k=1}^{m}a_{\mathrm{ik}}$$

where $\mu_{\mathrm{ij}}$ is the mean of the *z*‐scores in the ith row of matrix $A_{j}$ for subject j, $a_{\mathrm{ik}}$ denotes the *z*‐score in the ith row and kth column of matrix $A_{j}$, and m represents the number of regions. After calculating the mean for each row, we construct a mean vector $\mu_{j}$ for each subject j, where each element of the vector corresponds to the mean of a row in matrix $A_{j}$.

$$\mu_{j}=\left[\mu_{j1}\;\mu_{j2}\ldots \;\begin{array}{cc} \mu_{\mathrm{jm}-1} & \mu_{\mathrm{jm}} \end{array}\right]$$

Ultimately, we obtain a matrix RED of dimension s×m, where each row s corresponds to a subject, and each column m corresponds to a region.

$$\mathrm{RED}=\left[\begin{array}{ccc} \mu_{11} & \cdots & \mu_{1m}\\ \vdots & \ddots & \vdots \\ \mu_{s1} & \cdots & \mu_{\mathrm{sm}} \end{array}\right]$$

The element at position $\left(j,i\right)$ represents the mean deviation for region i in subject j. This metric assigns a deviation value for each region for each subject.

Both the SED and RED are then used to perform statistical comparisons of deviation patterns between groups.

### 2.4 Network Perturbation Approach (Application)

The dataset of 71 HCs was used as a reference cohort to construct a group‐level molecular network using the perturbation network approach. In this network, nodes corresponded to brain regions as defined by the Hammersmith atlas (Hammers et al. 2003), and the edges between nodes represented partial correlation coefficients of SUVr between brain regions, with age and sex included as covariates, since the FDOPA signal has been shown to be influenced by these factors (Kumakura et al. 2010; Laakso et al. 2002). Patients were then used to perturb the network and generate individual‐level deviation matrices. Additionally, for each patient group, subject‐level (SED) and regional level (RED) network metrics were extracted. Furthermore, the stability of the group‐level molecular network for the HCs was assessed through a resampling procedure (Lu et al. 2024). Groups of 8 to 64 subjects, incremented by 4, were randomly selected from the 71 available HCs. For each new group, a corresponding group‐level molecular network was constructed, and stability was evaluated by performing a correlation analysis between the newly constructed network and the original group‐level network. This resampling procedure was repeated 20 times for each group size.

### 2.5 Study 1: Parkinson's Disease and Schizophrenia

The objective of this study was to evaluate the efficacy of single‐subject networks in differentiating and characterizing PD and SCZ based on deviations in molecular covariance from normative patterns. Given the distinct pathophysiological mechanisms underlying these conditions—nigrostriatal dysfunction in PD (de Lau and Breteler 2006) and elevated presynaptic dopamine levels in SCZ (Howes et al. 2024)—we hypothesized that each condition would exhibit unique patterns of molecular connectivity alterations. Specifically, we anticipated that PD would display pronounced, localized deviations reflecting specific nigrostriatal pathology, whereas SCZ would present a more heterogeneous pattern, involving a broader range of regions associated with altered dopamine activity.

To test these hypotheses, single‐subject networks for PD and SCZ subjects were constructed using the HC group as the reference cohort. Extreme deviations at both the subject level (SED) and regional level (RED) were extracted following previously outlined methodologies.

Statistical comparisons of SED between the two groups were conducted using the Wilcoxon rank‐sum test, with the magnitude of group differences quantified by Cohen's d effect size. Subsequently, RED distributions across brain regions were analyzed using the same statistical framework, comparing PD and SCZ groups via the Wilcoxon rank‐sum test and Cohen's d. To assess regional significance, each brain region's RED was tested with a Wilcoxon rank‐sum test corrected for multiple comparisons using the false discovery rate (FDR, *p* < 0.05). The top 10% of regions with the highest mean RED were identified separately for the PD and SCZ groups.

In the final analysis, both vectorized lower triangular deviation matrices and RED values were used as features to classify SCZ versus PD. Classification was performed using a linear Support Vector Machine (SVM) with 5‐fold cross‐validation (CV) and an 80/20 training/test split. Model performance was evaluated using balanced accuracy (ACC), sensitivity, specificity, and the area under the receiver operating characteristic curve (AUC‐ROC) as performance metrics. For additional robustness, we performed 1000 permutations by randomly reshuffling the labels between the two groups to generate a null distribution. We then statistically compared the original balanced accuracy to the null distribution, obtaining an empirical *p*‐value.

### 2.6 Study 2: Psychosis Spectrum Disorders

In this second study, the primary objective was to determine whether a discernible gradient of molecular covariance deviations exists across the psychosis continuum, progressing from CHR to FEP and SCZ, and to assess whether this gradient could be effectively captured using single‐subject networks.

Statistical analyses were conducted using the Kruskal‐Wallis test to compare SED and RED across the three groups. Post hoc comparisons were performed using Dunn's test, corrected for multiple comparisons using the false discovery rate (FDR, *p* < 0.05), to identify significant differences between specific groups. To further evaluate RED, each brain region was assessed with a Wilcoxon rank‐sum test (FDR‐corrected, *p* < 0.05) to determine which regions exhibited statistically significant differences among the groups. As in the previous analysis, the top 10% of regions with the largest mean RED extreme deviations were identified separately for each group.

For classification, we employed the same machine learning approach as in the previous study, using a linear SVM with 5‐fold CV and an 80/20 training/test split. However, in this case, the model was adapted for multiclass classification using a one‐vs‐one strategy, and the Synthetic Minority Over‐sampling Technique (SMOTE) algorithm was used for class imbalance.

### 2.7 Study 3: FEP Responders and Non‐Responders

This study included the FEP subjects of study 2, for whom both baseline and follow‐up acquisitions were available (*n* = 18). The objective of this study was to evaluate whether single‐subject networks could detect differences in molecular deviation patterns between TR and non‐TR FEP subjects to antipsychotic medication at baseline. Additionally, we aimed to assess the impact of medication on the deviation pattern on TR between baseline and follow‐up acquisition. Based on prior literature (Jauhar et al. 2019), we hypothesized that baseline dopamine alterations would differ between TR and non‐TR groups, and that at follow‐up, antipsychotic medication would induce a significant decrease in molecular covariance alterations in TR subjects.

To test these hypotheses, a one‐tailed Wilcoxon signed‐rank test was used to compare SED between TR subjects at baseline and follow‐up. Additionally, a Wilcoxon rank‐sum test was employed to compare SED between TR and non‐TR subjects at baseline. Subsequently, the top 10% of regions with the largest mean RED at baseline in the TR group were identified. These regions were compared with the same regions at follow‐up in the TR group using a one‐tailed Wilcoxon signed‐rank test and with non‐TR subjects at baseline using a Wilcoxon rank‐sum test. These analyses aimed to provide insights into the potential effects of antipsychotic treatment on molecular covariance patterns and to explore differences in alteration patterns between TR and non‐TR groups.

Finally, we evaluated whether single‐subject matrices could serve as fingerprints to uniquely match baseline and follow‐up data for individual subjects. For each subject at baseline, the best match among n subjects at follow‐up was identified by calculating the Pearson correlation coefficient between the lower triangles of their individual molecular connectome matrices. Successful identification occurred when the best‐matched subject at follow‐up had the same identity as the baseline subject. The identification accuracy rate was calculated as the proportion of successful identifications out of the total number of identifications.

As a supplementary analysis, we tested the performance of this approach when applied to HC data acquired at both baseline and follow‐up. SED and RED were statistically compared using a Wilcoxon signed‐rank test to evaluate whether the method could accurately return no statistical difference between data from the same subject acquired at different time points.

## 3 Results

The preliminary stability analysis of the reference group size indicated that when the number of HCs was equal to or exceeded 36, stability—measured by the correlation coefficient—consistently remained above 0.9 with minimal variance. These findings suggest that the reference matrix is robust even when using a relatively smaller reference group of HCs.

### 3.1 Study 1: PD Vs SCZ

**FIGURE 2:**
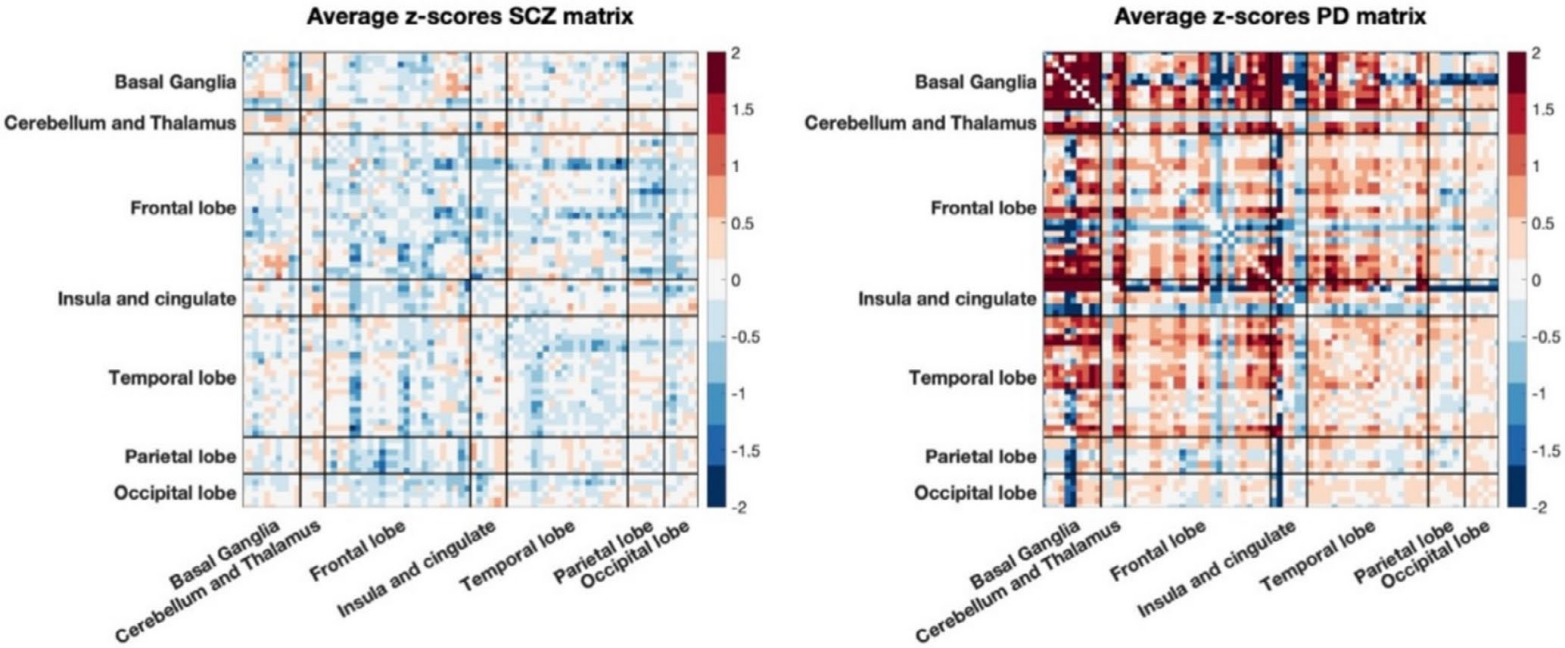
Average of individual deviation matrices across subjects for SCZ group (left) and for PD group (right).

**FIGURE 3:**
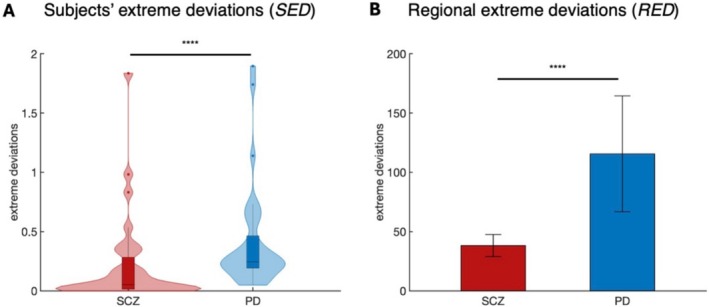
Statistical comparison of extreme deviations between PD and SCZ. (A) Results of the Wilcoxon rank‐sum test for subject‐level extreme deviations (SED). (B) Results of the Wilcoxon rank‐sum test of regional‐level extreme deviations (RED). Asterisks denote significance levels: ****p < 0.0001, ***p < 0.001, **p < 0.01, *p < 0.05.

In Figure 2 are reported the average of the individual deviations' matrices for SCZ and PD respectively. To test the hypothesis that PD and SCZ subjects exhibit distinct network deviations relative to HCs—both at the whole‐subject level and across regions—we conducted statistical analyses of SED and RED. The analyses revealed significant differences between the two conditions for both SED (*p* < 0.001, Cohen'*d* = 0.45) (Figure 3A) and RED (*p* < 0.0001, Cohen'*d* = 0.54) (Figure 3B), confirming that the overall magnitude and spatial distribution of deviations vary between PD and SCZ.

**FIGURE 4:**
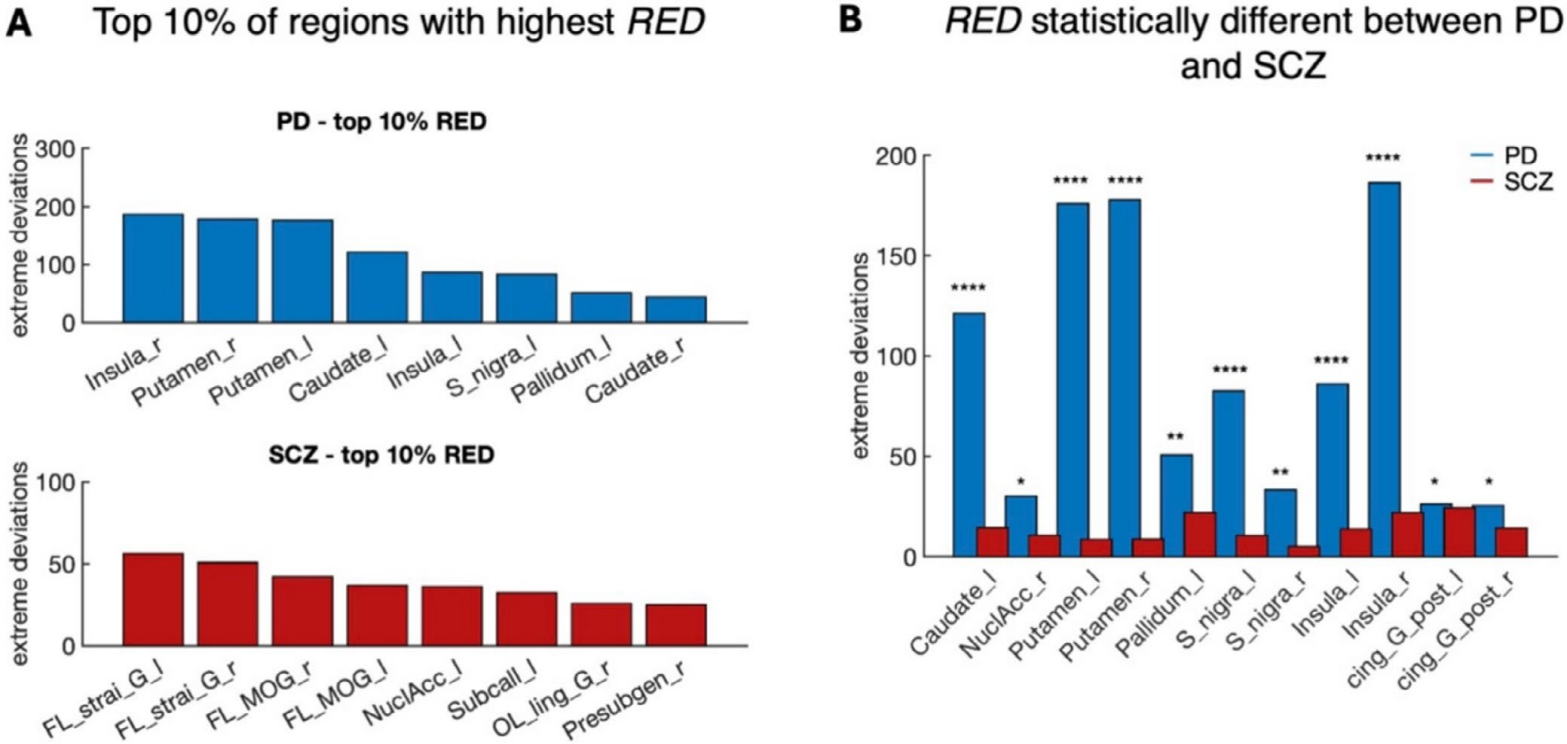
(A) Top 10% of regions with the highest RED for both PD (top) and SCZ (bottom). (B) Regions with statistically significant differences between PD and SCZ (Wilcoxon rank‐sum test, FDR‐corrected, *p* < 0.05). Asterisks denote significance levels: ****p < 0.0001, ***p < 0.001, **p < 0.01, *p < 0.05. [left hemisphere (l), right hemisphere (r), frontal lobe (FL), occipital lobe (OL), straight gyrus (strai_G), medial orbital gyrus (MOG), lingual gyrus (ling_G), cingulate gyrus posterior part (cing_G_post), pre‐subgenual (Presubgen)].

Further analysis of the top 10% of regions with the highest mean RED revealed distinct condition‐specific patterns. In PD, the largest deviations were localized to regions associated with neurodegeneration, including the insula, basal ganglia, and substantia nigra (Figure 4A). In contrast, SCZ exhibited subtler deviations, predominantly involving the prefrontal cortex, limbic system and ventral striatum (Figure 4A). These findings provide strong evidence that PD and SCZ are characterized by unique spatial patterns of network alterations, consistent with the divergent pathophysiology underlying these disorders.

To pinpoint specific regions with significantly different deviations between PD and SCZ, we performed region‐wise statistical tests. Following FDR correction (*p* < 0.05) significant differences were identified in the basal ganglia, substantia nigra, and posterior cingulate cortex (Figure 4B). These regions emerged as key contributors to the divergence in network patterns between PD and SCZ, emphasizing their relevance in disease‐specific pathophysiological processes.

**FIGURE 5:**
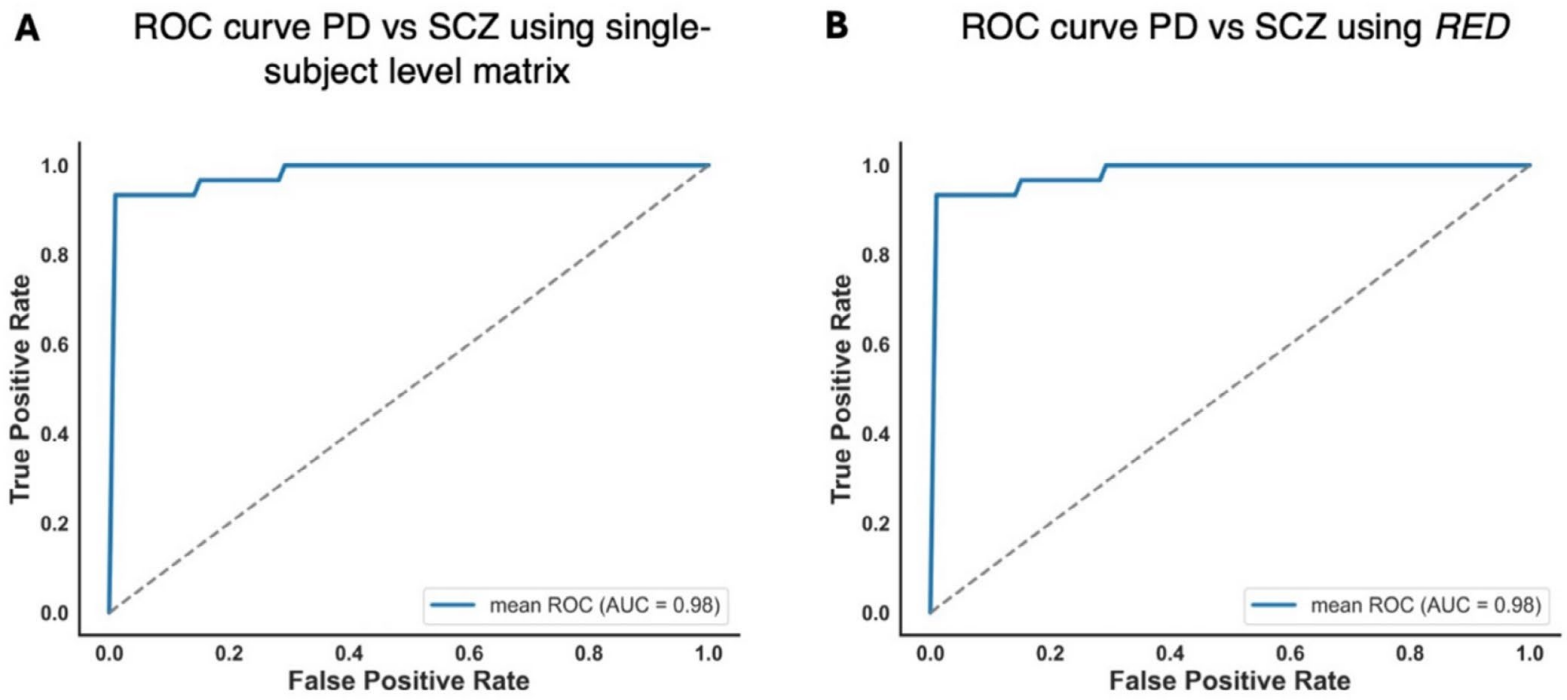
ROC and AUC for the classification of PD and SCZ groups. (A) ROC curve and AUC for the classification of PD and SCZ groups using vectorized lower triangular single‐subject matrices. (B) ROC curve and AUC for the classification of PD and SCZ groups using RED.

Finally, to evaluate the potential of RED and individual deviation matrices as features for distinguishing PD and SCZ, we trained a machine learning classifier using a linear SVM. The classifier demonstrated excellent performance, both when using vectorized lower triangular matrices (ACC = 94%, AUC = 0.99, sensitivity = 0.96, specificity = 0.91) (Figure 5A) and RED as features (ACC = 94%, AUC = 0.98, sensitivity = 0.9, specificity = 0.97) (Figure 5B). These results further highlight how single subject level matrices could perfectly characterize the divergent nature of these two brain disorders taking into consideration individual heterogeneity. Furthermore, the comparison with the null distribution of randomly permuted labels revealed a statistically significant difference (*p* < 0.001).

### 3.2 Study 2: Psychosis Continuum

**FIGURE 6:**
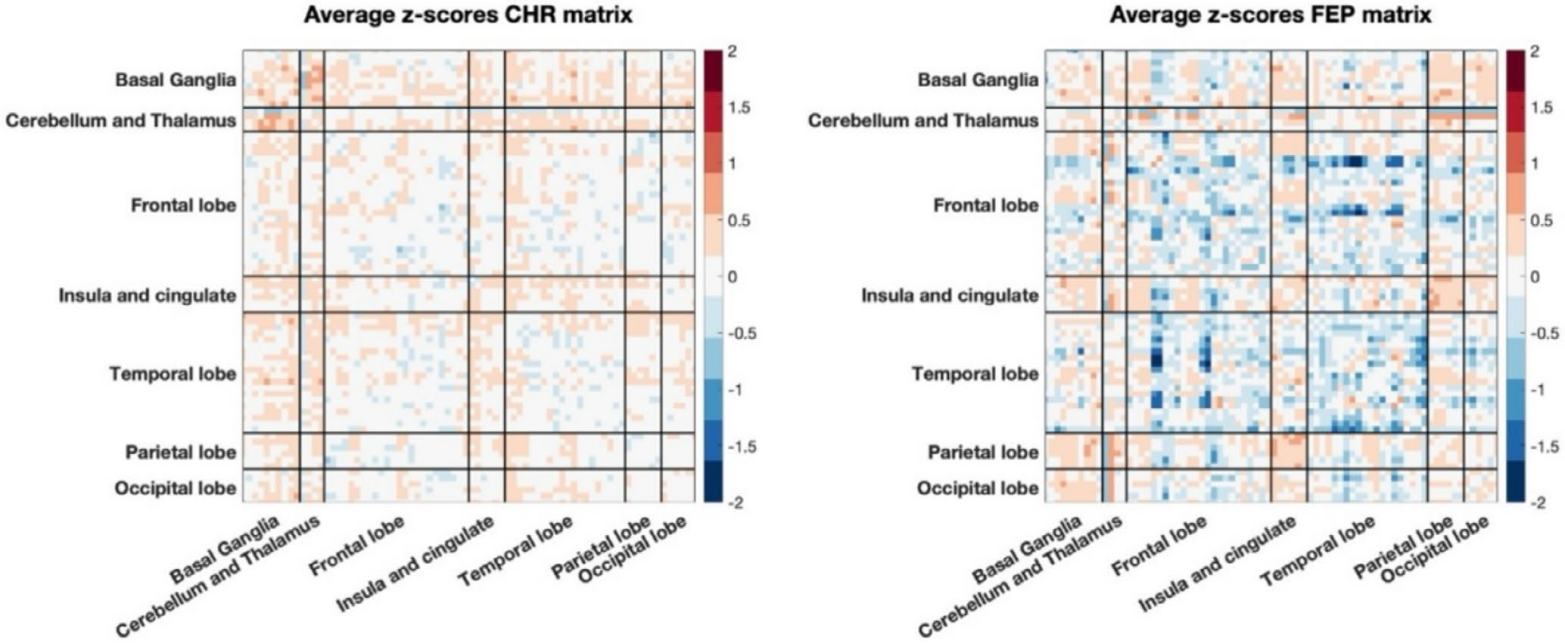
Average of individual deviation matrices across subjects for CHR group (left) and for FEP group (right).

In Figure 6 are reported the average of the individual deviations' matrices for CHR and FEP respectively. To investigate whether a gradient in network deviations could be discerned across the psychosis spectrum, we statistically compared groups of individuals at CHR, those with FEP, and SCZ using both SED and RED.

**FIGURE 7:**
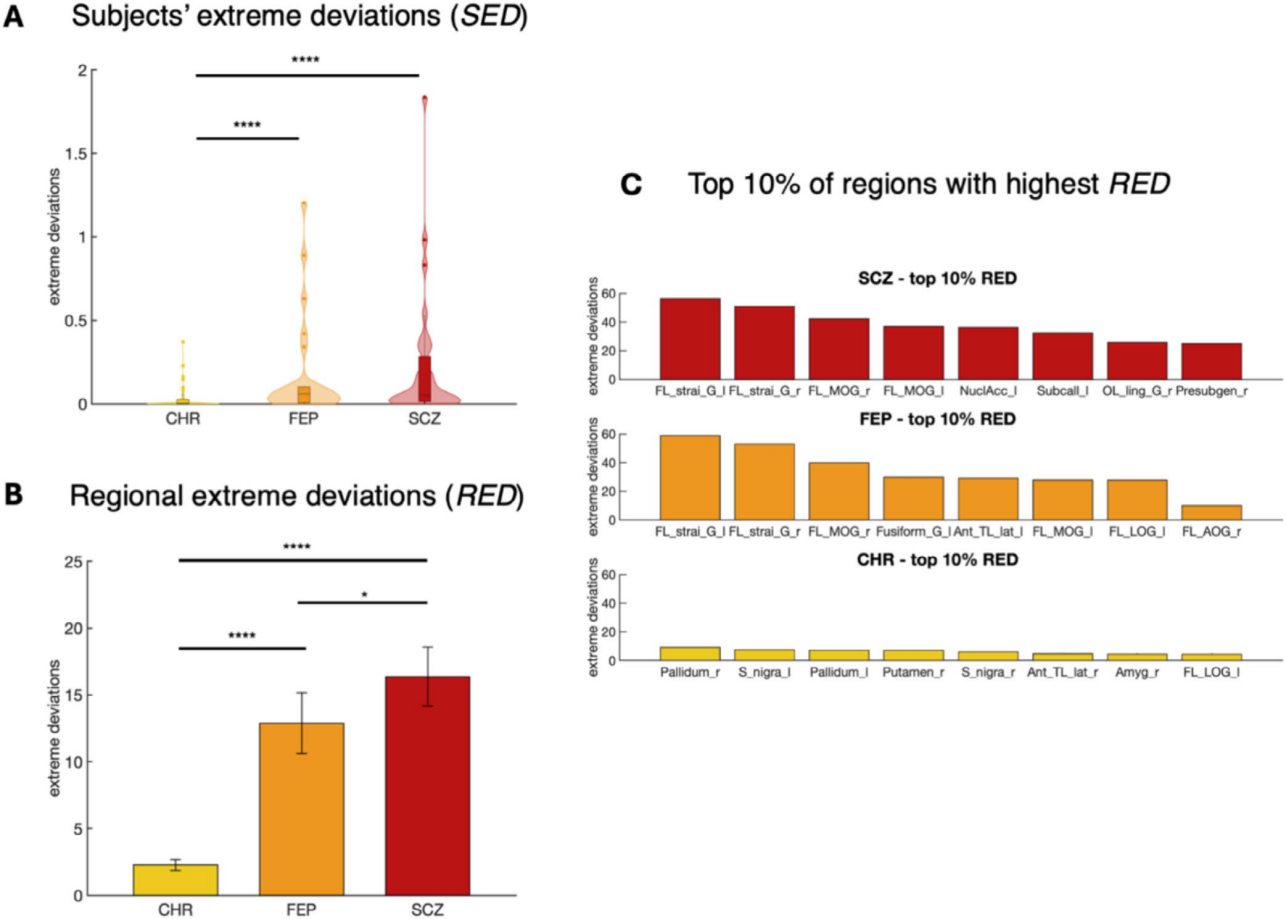
Statistical comparison of extreme deviations between CHR, FEP and SCZ. (A) Results of SED comparison between CHR, FEP, and SCZ using the Dunn post hoc test (FDR‐corrected, *p* < 0.05). (B) Results of RED comparison between CHR, FEP, and SCZ using the Dunn post hoc test (FDR‐corrected, *p* < 0.05) (*RED*). (C) Top 10% of regions with highest RED for SCZ (top), FEP (middle) and CHR (bottom). Asterisks denote significance levels: ****p < 0.0001, ***p < 0.001, **p < 0.01, *p < 0.05. [left hemisphere (l), right hemisphere (r), frontal lobe (FL), temporal lobe (TL), straight gyrus (strai_G), medial orbital gyrus (MOG), anterior orbital gyrus (AOG), lateral orbital gyrus (LOG), anterior temporal lobe lateral part (Ant_TL_lat)].

The Kruskal‐Wallis test applied to SED revealed significant differences among the three groups (*χ* ^2^ = 20.6, *p* < 0.0001). Post hoc Dunn's tests with FDR correction confirmed significant deviations between the CHR and SCZ groups (*p* < 0.0001, Cohen'*d* = 0.85) and between the CHR and FEP groups (*p* < 0.01, Cohen'*d* = 0.75). However, no significant differences were observed between the FEP and SCZ groups (*p* = 0.85, Cohen'*d* = 0.12), suggesting that these two diagnostic categories exhibit comparable distributions of overall deviations (Figure 7A).

Similarly, the analysis of RED revealed significant group differences (*χ* ^2^ = 143.4, *p* < 0.0001) in the Kruskal‐Wallis test. Pairwise comparisons demonstrated significant deviations between all groups, CHR and FEP (*p* < 0.0001, Cohen'*d* = 1.4), CHR and SCZ (*p* < 0.0001, Cohen'*d* = 2) and FEP and SCZ (*p* = 0.04, Cohen'*d* = 0.35) (Figure 7B). Subtle differences were observed in the top 10% of regions with the highest mean RED, where FEP exhibited slightly distinct regions compared to SCZ. However, these regions were similarly concentrated in the frontal lobe, particularly in the orbital gyri (Figure 7C).

When testing for specific regions that differed significantly among the three diagnostic groups, no regions survived multiple comparison correction in the FEP versus SCZ comparisons. In contrast, nearly all regions exhibited significant differences when comparing CHR to SCZ. Additionally, significant differences were observed across all regions of the frontal lobe, temporal lobe, occipital lobe, and cingulate cortex when comparing CHR to FEP. These findings highlight pronounced differences across all regions between preclinical and clinical conditions.

**FIGURE 8:**
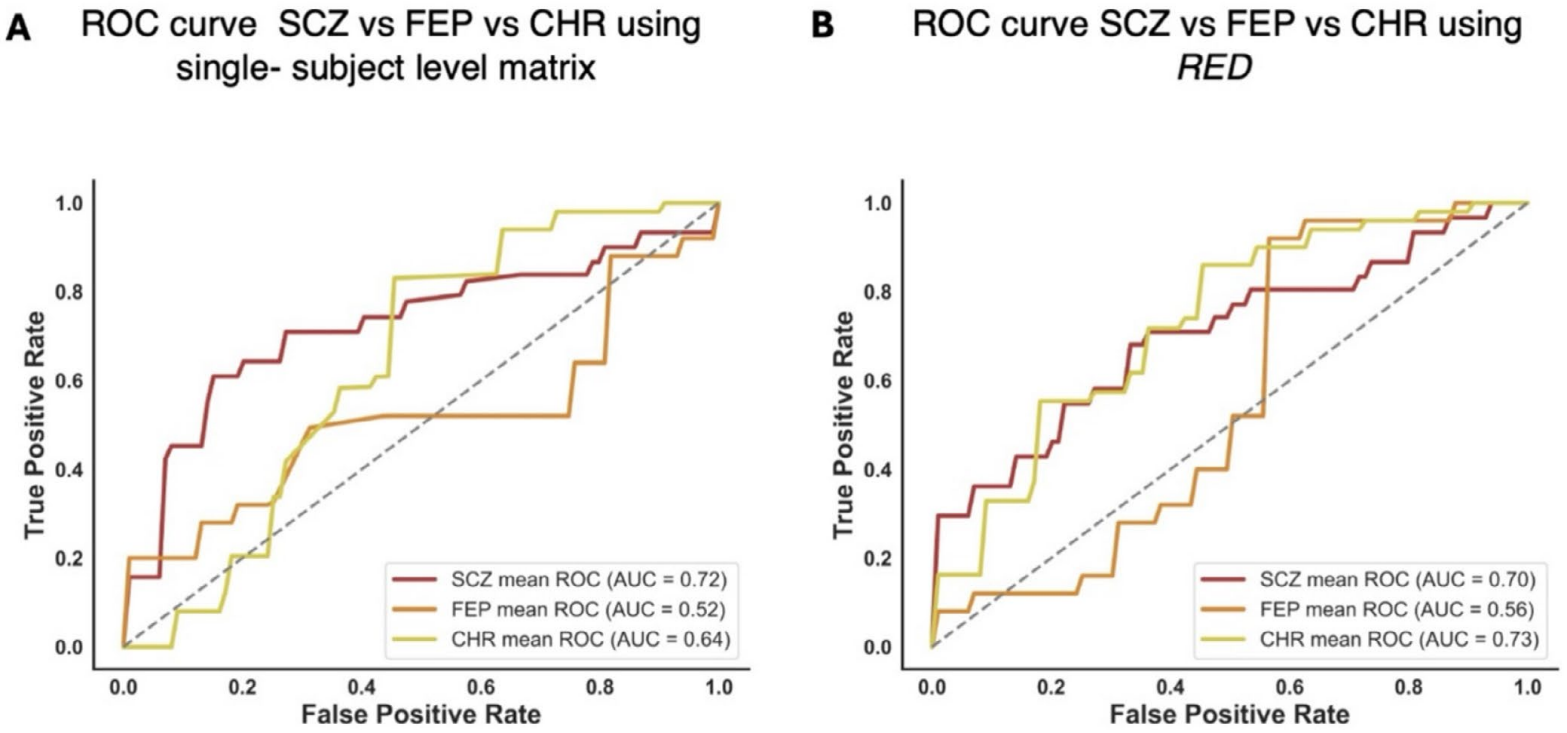
ROC and AUC for the classification of CHR, FEP and SCZ groups. (A) ROC curve and AUC for the classification of CHR, FEP and SCZ groups using vectorized lower triangular single‐subject matrices. (B) ROC curve and AUC for the classification of CHR, FEP and SCZ groups using RED.

A machine learning model was trained to classify CHR, FEP, and SCZ groups based on deviation patterns. Using vectorized lower triangular matrices as input, the model achieved moderate performance (ACC = 45.5%, average AUC = 0.66, average sensitivity = 0.45 and average specificity = 0.76) (Figure 8A). Slightly better results were obtained using RED (ACC = 52.1%, average AUC = 0.6, average sensitivity = 0.51 and average specificity = 0.76) (Figure 8B).

These results exceeded the chance‐level accuracy of 33.3%, but also revealed challenges in the classifier's ability to accurately categorize certain subjects across the three groups. This difficulty stemmed from the classifier's struggle to identify distinct subject‐level patterns that could reliably differentiate the groups. The overlap in alteration patterns among subjects at intermediate stages of the spectrum reflects this challenge. These findings highlight the high heterogeneity within the groups, where individuals within the same group may exhibit markedly different deviation patterns, complicating the precise assignment to a specific stage.

### 3.3 Study 3: Treatment Response in Psychosis

**FIGURE 9:**
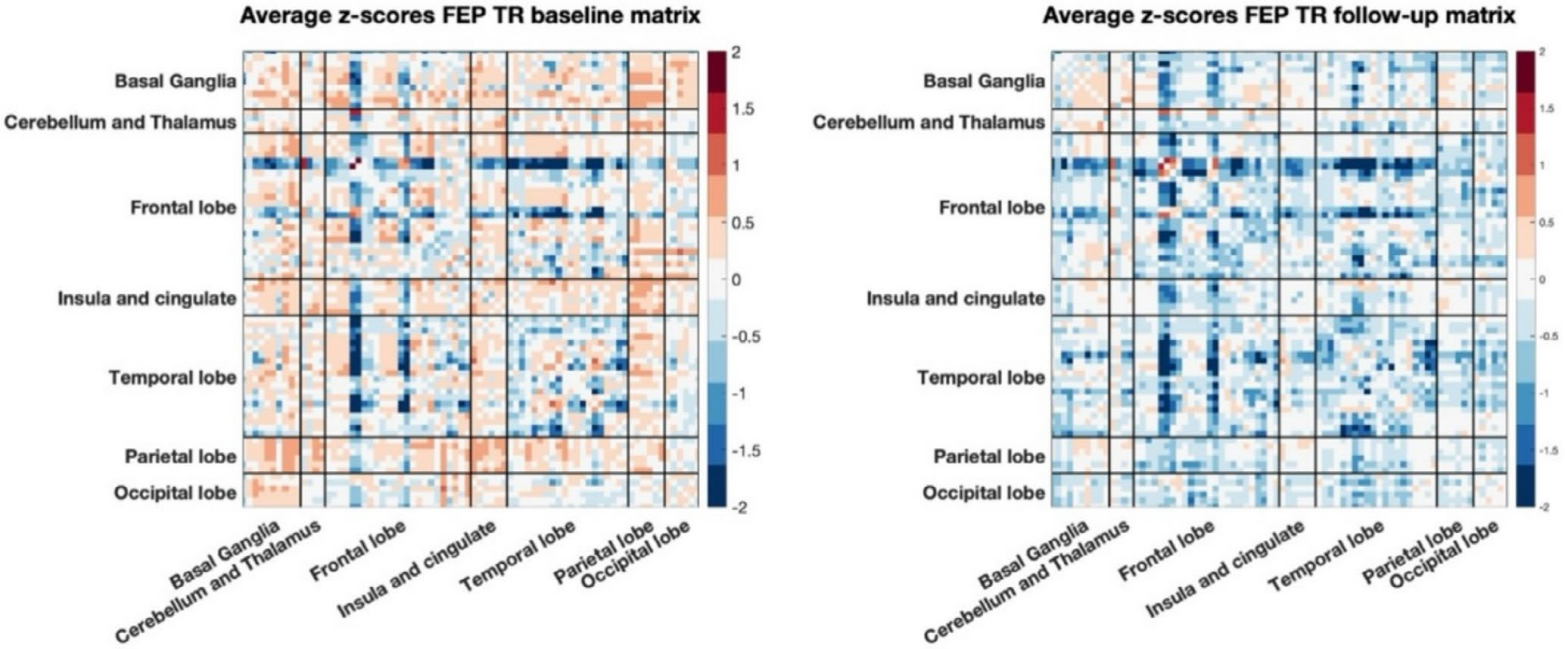
Average of individual deviation matrices across subjects for FEP TR at baseline (left) and at follow‐up (right).

**FIGURE 10:**
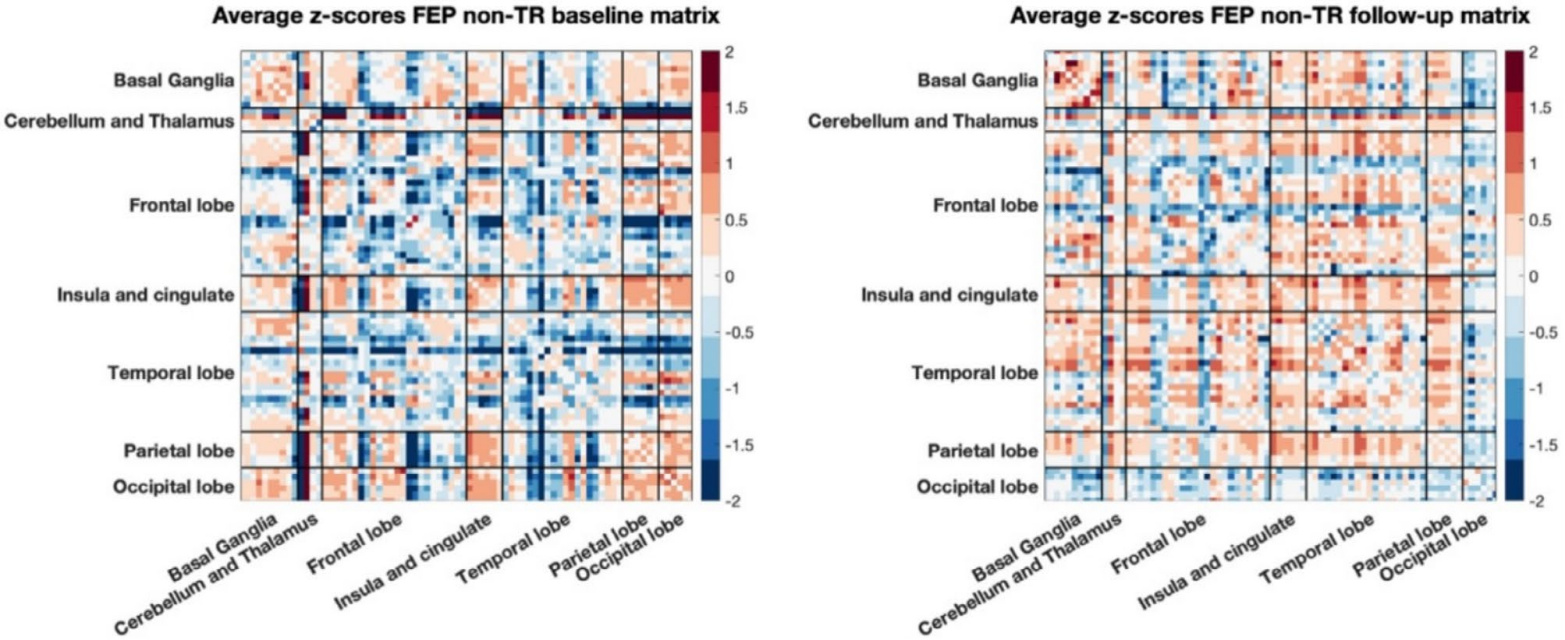
Average of individual deviation matrices across subjects for FEP non‐TR at baseline (left) and at follow‐up (right).

In Figure 9 are reported the average of the individual deviations' matrices for FEP TR at baseline and follow‐up respectively while in Figure 10 are reported for FEP non‐TR at baseline and follow‐up. To explore whether deviations in molecular connectivity could differentiate between TR and non‐TR in FEP, we analyzed SED and RED metrics. Additionally, we investigated the stability and uniqueness of molecular connectomes over time using an individual network fingerprinting approach.

The analysis of SED showed no significant statistical differences between FEP TR and non‐TR subjects at baseline (*p* = 0.46). Similarly, no significant decrease in subject‐level alterations was observed in FEP TR between baseline and follow‐up (*p* = 0.36), suggesting that the overall proportion of deviations does not effectively differentiate between these groups.

**FIGURE 11:**
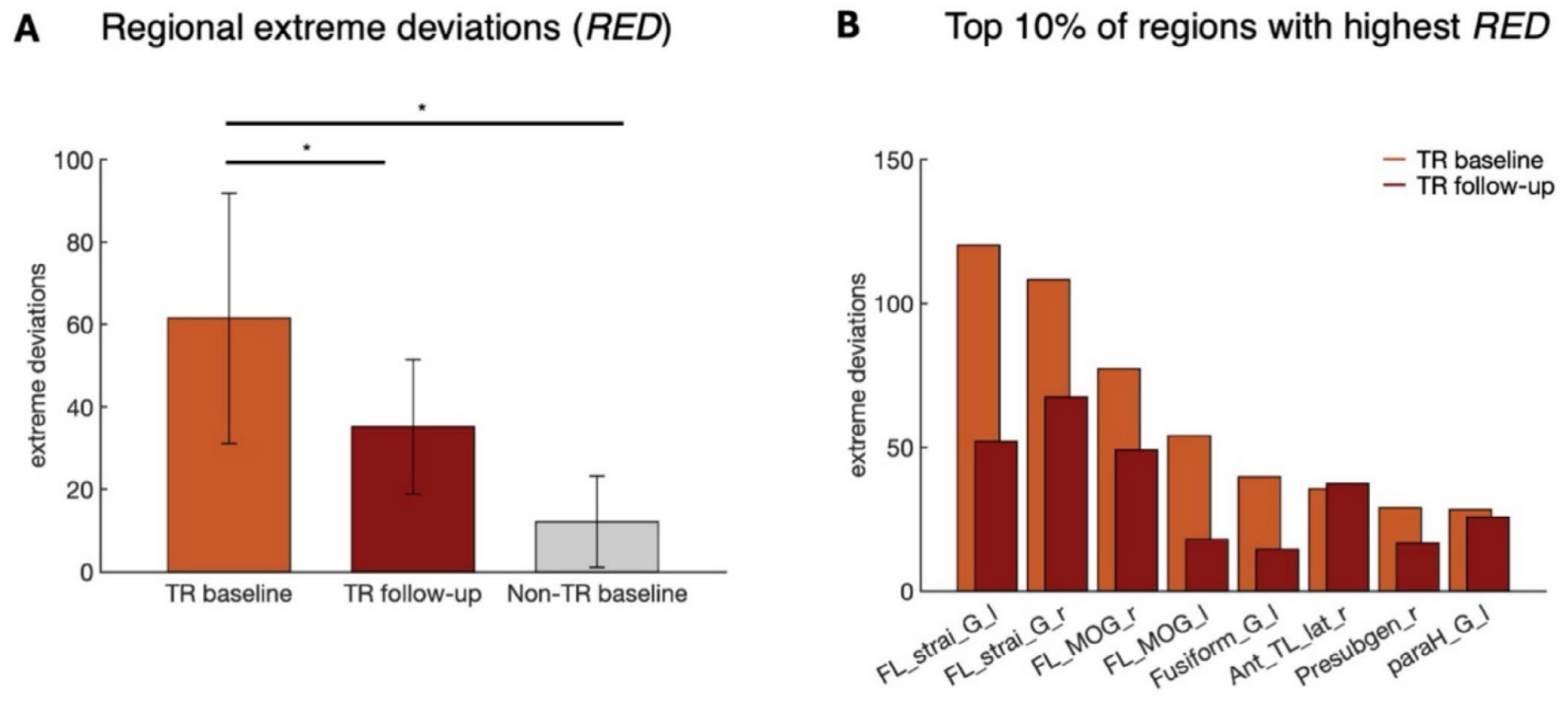
Statistical comparison of the top 10% of regions with the highest RED in TR baseline compared to the same regions in TR at follow‐up and non‐TR at baseline. (A) Results of one‐tailed Wilcoxon signed‐rank test between TR at baseline and follow‐up, and Wilcoxon rank‐sum test between TR at baseline and non‐TR at baseline. (B) Comparison of the top 10% of regions with the highest RED in TR at baseline and their corresponding values at follow‐up. Asterisks denote significance levels: ****p < 0.0001, ***p < 0.001, **p < 0.01, *p < 0.05. [left hemisphere (l), right hemisphere (r), frontal lobe (FL), straight gyrus (strai_G), medial orbital gyrus (MOG), parahippocampal and ambient gyri (paraH_G), anterior temporal lobe lateral part (Ant_TL_lat), pre‐subgenual (Presubgen)].

In contrast, analysis of the top 10% of regions with the highest RED revealed significant differences consistent with our hypotheses. At baseline, FEP TR subjects exhibited significantly different regional deviation patterns compared to non‐TR (*p* = 0.03, Cohen'*d* = 0.94) (Figure 11A). These findings indicate that the molecular regional deviations associated with antipsychotic treatment response differ from those observed in non‐responders. Furthermore, a significant reduction in regional deviations was detected in TR at follow‐up compared to baseline (*p* = 0.02, Cohen'*d* = 0.75) (Figure 11A), suggesting that pharmacological treatment influences regional deviation patterns. These results highlight differences in the molecular regional deviations of the FDOPA signal between FEP subjects likely to respond to antipsychotic treatment and non‐responders. Additionally, the reduction in regional deviations at follow‐up in TR suggests that antipsychotic treatment induces measurable changes in molecular connectivity patterns over time (Figure 11B).

To assess whether individual molecular connectomes could reliably identify subjects over time, we conducted a network fingerprinting analysis. This approach demonstrated high classification accuracy for identifying individuals within both diagnostic groups (ACC = 83.3% for TR and ACC = 83.3% also for non‐TR). These findings suggest that the influence of pharmacological treatment primarily manifests as a reduction in the magnitude of deviations, rather than as a reorganization of the underlying topological pattern. Consequently, the similarity between an individual's baseline and follow‐up connectivity patterns remains higher than the similarity observed between different individuals.

## 4 Discussion

The present study introduced a framework that applies, for the first time, covariance perturbation principles to analyze static FDOPA PET data. This framework provides a straightforward, implementable methodology for identifying molecular differences between brain regions at the single‐subject level.

Consistent with our hypotheses, the subject‐level deviation matrices generated by the proposed framework effectively captured divergent molecular alteration patterns in PD relative to SCZ at the individual level. Additionally, the framework identified a progressive increase in alterations corresponding to advancing disease stages in the psychosis spectrum. The regional alterations also differed between FEP TR and non‐TR, suggesting distinct FDOPA alteration patterns, in line with previous findings from FDOPA univariate analysis (Veronese et al. 2021; Jauhar et al. 2019). Furthermore, the regional alterations were significantly lower in TR subjects at follow‐up, indicating potential effects of antipsychotic treatment in modulating these alterations. Finally, the single‐subject networks successfully captured individual‐specific characteristics through a fingerprinting approach, enabling the identification of individual patients across baseline and follow‐up.

Interestingly, when looking at the single subjects' networks, PD subjects predominantly exhibited positive deviations (*z*‐scores), suggesting that the addition of data from an individual with PD strengthens the correlation within the reference matrix. In contrast, subjects within the psychosis spectrum demonstrated a predominance of negative deviations, indicating that the inclusion of data from an individual with psychosis weakens the correlation pattern between brain regions. While the biological interpretation of these deviations is likely complex, these findings underscore the divergent directions of molecular network alterations in PD and psychosis spectrum disorders. To further explore these deviation patterns, metrics were derived from single‐subject networks to quantify the proportion of extreme deviations both at the individual subject level and within specific brain regions for each subject.

The decision to threshold the networks to isolate extreme deviations enabled us to retain only those deviations significant for discrimination purposes, effectively filtering out low‐magnitude deviations likely attributable to noise. Our selected threshold corresponds to a significance level of < 0.05 Bonferroni corrected. However, we also tested with different thresholds, ranging from 2 (no correction) to 4.13 (Bonferroni correction), and the SED and RED metrics remained consistent, consistently showing a correlation above 0.9 across all thresholds. Finally, both deviation edges and the RED proved to be effective features in machine learning models, yielding good discrimination performance between subjects from different groups. These findings demonstrate that the deviation patterns effectively capture subject heterogeneity, underscoring their potential for group differentiation.

The principal strengths of this method lie in its ability to account for inter‐subject heterogeneity and provide a comprehensive examination of molecular interactions and alterations across brain regions at a systems level. Each region's alteration, representing its anomalous connection relative to other regions in comparison to a normative cohort, can be effectively quantified. Unlike conventional approaches that often focus on specific regions, this methodology enables the exploration of molecular alterations across the entire brain. This holistic approach reveals deviation patterns in extrastriatal regions that are typically overlooked in traditional FDOPA analyses, which primarily emphasize the striatum. FDOPA is not a specific ligand for dopamine neurons but is trapped by all neurons containing the enzyme aromatic amino acid decarboxylase (AADC). Consequently, it serves as a marker for all tissues that take up and store monoamines, including serotonin, noradrenaline, and dopamine neurons (Moore et al. 2008; Brown et al. 1999). AADC is thought to play a role in psychiatric disorders (Sedvall and Farde 1995), limbic functions (Dolan et al. 1995), and the general modulation of cerebral activity, as well as in parkinsonism. This broader functionality suggests that FDOPA can serve as an indicator of neuronal loss not only in dopaminergic regions but also in areas containing noradrenergic or serotonergic cell bodies or terminals (Brown et al. 1999; Moore et al. 2003). Therefore, regions outside the striatum may also contribute to the signal detected with this radiotracer.

Indeed, several extrastriatal regions exhibited a high proportion of deviations from normative values in the patient cohorts, both in individuals with SCZ and FEP and also in PD. Among these, the insula emerged as the region showing the largest deviation outside the striatum in PD, and in a lower magnitude it was deviated also in SCZ. The insula is significantly affected in PD due to alpha‐synuclein deposition, altered neurotransmitter function, disruptions in connectivity, and concomitant metabolic and structural abnormalities (Criaud et al. 2016). Furthermore, another study pointed out the insula's critical role as an integrative hub, responsible for processing interoceptive signals and integrating cognitive‐affective, sensorimotor, and autonomic information, and is likely compromised in PD, leading to widespread functional impairments (Christopher et al. 2014). In SCZ, disruptions in insular function have also been documented, highlighting its involvement in sensory‐affective processing deficits and its broader implications for the pathophysiology of psychosis (Wylie and Tregellas 2010). Moreover, the insula also forms part of the salience network, which is altered in SCZ (Menon et al. 2023).

Additional deviated extrastriatal regions, spanning the prefrontal cortex, limbic system, temporal lobe, and occipital lobe, were particularly pronounced in the SCZ and FEP groups. These deviations are consistent with neuroanatomical studies that have reported marked grey matter volume reductions in the frontal, temporal, limbic, and parietal lobes in individuals with psychotic disorders (Adamu et al. 2023). Specifically, volumetric changes in the occipital lobe (Tohid et al. 2015), prefrontal cortex, and medial temporal lobe have been associated with deficits in working memory and declarative memory functions (Karlsgodt et al. 2010). Moreover, structural anomalies in the superior temporal gyrus, along with disrupted white matter connectivity in the temporal and frontal lobes, have also been implicated in the neurobiology of psychosis (DeLisi et al. 2006). Finally, a meta‐analysis by the ENIGMA consortium, involving thousands of participants, found thinner cortical regions in subjects with SCZ compared to HC. The largest effect sizes were observed in the frontal and temporal lobes (van Erp et al. 2018).

It is important to recognize that our method does not produce a direct molecular connectivity network for individual subjects, but a perturbation network referenced against a normative cohort. This framework captures deviations in molecular connectivity by contrasting normal and pathological samples, thus highlighting regions of divergence rather than constructing an absolute connectivity map. Consequently, the method relies on the availability of a robust and representative reference group to ensure reliable and meaningful analysis. This approach is analogous to normative modeling (Marquand et al. 2016), where its primary strength lies in evaluating group‐level differences relative to a reference cohort.

### 4.1 Limitations

Several findings from the present study require cautious interpretation due to certain methodological limitations. First, our analysis was primarily limited to cross‐sectional comparisons, which constrain our ability to infer temporal dynamics or longitudinal patterns in the observed deviation trajectories. Incorporating more longitudinal data would offer a more comprehensive understanding of the progression of molecular network alterations over time. Second, the analysis focused exclusively on extreme deviations in absolute terms, without considering the directionality of these deviations. This choice was made due to the complexity of interpreting the sign of deviations within individual molecular connectomes, where the direction reflects statistical divergence from the reference model rather than a clear functional deviation linked to specific biological processes. As a result, this methodological approach may have obscured subtle but biologically significant directional shifts in molecular connectivity patterns.

Another important limitation is the potential sensitivity of our individual network analysis to variations in scanning protocols. Such variations could introduce deviations from the reference cohort attributable to extraneous factors unrelated to disease processes or therapeutic effects (Veronese et al. 2019). This highlights the need for rigorous validation of the methodology to ensure its reliability. Variability in scanning conditions could limit the generalizability of our findings, potentially affecting the reproducibility of results across diverse cohorts and imaging environments (Veronese et al. 2019).

A larger sample size for the classification tasks in Study 2 would have allowed for more robust and reliable conclusions, thereby enhancing the generalizability of the findings.

Another potential limitation concerns the impact of pharmacological treatment. The FEP cohort was the only group with minimal or no treatment at the time of FDOPA PET scanning, while all other patient cohorts had received medication as part of standard clinical care. This may have influenced the FDOPA signal.

Finally, the exact pathophysiological significance of the networks analyzed in this study remains unclear. Future mechanistic investigations are needed to better understand the biological relevance of abnormalities in global network connectivity and regional alterations.

## 5 Conclusions

This study introduced a novel framework for constructing individual molecular abnormality networks, tested using static FDOPA PET imaging. This framework enabled a detailed characterization of FDOPA connectivity deviations by comparing individual molecular profiles to a reference cohort. By adopting a network‐based perspective, it complements conventional analytical methods by offering new insights into regions influenced by monoamine storage, which are typically overlooked in conventional FDOPA‐based analyses, and extends our understanding beyond the traditionally studied striatal regions.

The application of this framework holds significant promise for advancing the understanding of disease mechanisms, providing a unique lens to investigate the molecular underpinnings of neurological and psychiatric disorders. It also offers potential for evaluating the impact of pharmacological interventions and therapeutic strategies on molecular connectivity. While this framework demonstrates strong potential, further research is essential to assess its broader applicability across various clinical domains and conditions. Validation in larger and more diverse patient populations will help to determine its generalizability.

## Ethics Statement

All the research protocols for data acquisitions were approved by local ethics committees and local institutional review boards. Full details on approval protocol numbers are reported in the original references (Nordio et al. 2023; Li et al. 2018; Egerton et al. 2021, 2013; Jauhar et al. 2019). Informed written consent was obtained from all the participants, and the studies were conducted following the Declaration of Helsinki and Good Clinical Practice.

## Conflicts of Interest

Oliver Howes has received investigator‐initiated research funding from and/or participated in advisory/speaker meetings organized by Abbvie, Alkermes, Angellini, Autifony, Biogen, Boehringer‐Ingelheim, Delix, Eli Lilly, Elysium, Heptares, Global Medical Education, Invicro, Janssen, Karuna, Lundbeck, Merck, Neumora, Neurocrine, Ontrack/Pangea, Otsuka, Sunovion, Teva, Recordati, Roche, Rovi, and Viatris/Mylan. He was previously a part‐time employee of Lundbeck A/v. Oliver Howes and Mattia Veronese have a patent for the use of dopaminergic imaging. Robert McCutcheon has received speaker/consultancy fees from Boehringer Ingelheim, Janssen, Karuna, Lundbeck, Newron, Otsuka, and Viatris, and co‐directs a company that designs digital resources to support the treatment of mental ill health. The other authors declare no conflicts of interest.

## Data Availability

The data that support the findings of this study are available from The NeurOimaging DatabasE (NODE) repository (https://maudsleybrc.nihr.ac.uk/research/precision‐psychiatry/neuroimaging/neuroimaging‐database‐node/) but restrictions apply to the availability of these data, which were used under license for the current study, and so are not publicly available. Data are however available from the authors upon reasonable request and with permission by the data controller institutions, by contacting the support team (node.information@kcl.ac.uk) or M.V. (mattia.veronese@kcl.ac.uk). The code will be made available upon reasonable request.

## References

[hbm70253-bib-0001] Adamu, M. J. , L. Qiang , C. O. Nyatega , et al. 2023. “Unraveling the Pathophysiology of Schizophrenia: Insights From Structural Magnetic Resonance Imaging Studies.” Frontiers in Psychiatry 14: 1188603. 10.3389/fpsyt.2023.1188603. 37275974 PMC10236951

[hbm70253-bib-0002] Agid, O. , S. Kapur , T. Arenovich , and R. B. Zipursky . 2003. “Delayed‐Onset Hypothesis of Antipsychotic Action.” Archives of General Psychiatry 60, no. 12: 1228–1235. 10.1001/archpsyc.60.12.1228. 14662555

[hbm70253-bib-0003] 1991. “Dopamine in Schizophrenia: A Review and Reconceptualization.” American Journal of Psychiatry 148, no. 11: 1474–1486. 10.1176/ajp.148.11.1474. 1681750

[hbm70253-bib-0004] Bloomfield, M. A. P. , C. J. A. Morgan , A. Egerton , S. Kapur , H. V. Curran , and O. D. Howes . 2014. “Dopaminergic Function in Cannabis Users and Its Relationship to Cannabis‐Induced Psychotic Symptoms.” Biological Psychiatry 75, no. 6: 470–478. 10.1016/j.biopsych.2013.05.027. 23820822

[hbm70253-bib-0005] Brown, W. D. , M. D. Taylor , A. D. Roberts , et al. 1999. “FluoroDOPA PET Shows the Nondopaminergic As Well As Dopaminergic Destinations of Levodopa.” Neurology 53, no. 6: 1212–1218. 10.1212/WNL.53.6.1212. 10522875

[hbm70253-bib-0006] Calne, D. B. , J. W. Langston , W. R. W. Martin , et al. 1985. “Positron Emission Tomography After MPTP: Observations Relating to the Cause of Parkinson's Disease.” Nature 317, no. 6034: 246–248. 10.1038/317246a0. 3876510

[hbm70253-bib-0007] Christopher, L. , Y. Koshimori , A. E. Lang , M. Criaud , and A. P. Strafella . 2014. “Uncovering the Role of the Insula in Non‐Motor Symptoms of Parkinson's Disease.” Brain 137, no. 8: 2143–2154. 10.1093/brain/awu084. 24736308 PMC4107733

[hbm70253-bib-0008] Criaud, M. , L. Christopher , P. Boulinguez , et al. 2016. “Contribution of Insula in Parkinson's Disease: A Quantitative Meta‐Analysis Study.” Human Brain Mapping 37, no. 4: 1375–1392. 10.1002/hbm.23109. 26800238 PMC4874784

[hbm70253-bib-0009] de Lau, L. M. , and M. M. Breteler . 2006. “Epidemiology of Parkinson's Disease.” Lancet Neurology 5, no. 6: 525–535. 10.1016/S1474-4422(06)70471-9. 16713924

[hbm70253-bib-0010] DeLisi, L. E. , K. U. Szulc , H. C. Bertisch , M. Majcher , and K. Brown . 2006. “Understanding Structural Brain Changes in Schizophrenia.” Dialogues in Clinical Neuroscience 8, no. 1: 71–78. 10.31887/DCNS.2006.8.1/ldelisi. 16640116 PMC3181763

[hbm70253-bib-0011] Dolan, R. J. , P. Fletcher , C. D. Frith , K. J. Friston , R. S. J. Frackowiak , and P. M. Grasby . 1995. “Dopaminergic Modulation of Impaired Cognitive Activation in the Anterior Cingulate Cortex in Schizophrenia.” Nature 378, no. 6553: 180–182. 10.1038/378180a0. 7477319

[hbm70253-bib-0012] Egerton, A. , C. A. Chaddock , T. T. Winton‐Brown , et al. 2013. “Presynaptic Striatal Dopamine Dysfunction in People at Ultra‐High Risk for Psychosis: Findings in a Second Cohort.” Biological Psychiatry 74, no. 2: 106–112. 10.1016/j.biopsych.2012.11.017. 23312565

[hbm70253-bib-0013] Egerton, A. , A. Demjaha , P. McGuire , M. A. Mehta , and O. D. Howes . 2010. “The Test–Retest Reliability of 18F‐DOPA PET in Assessing Striatal and Extrastriatal Presynaptic Dopaminergic Function.” NeuroImage 50, no. 2: 524–531. 10.1016/j.neuroimage.2009.12.058. 20034580 PMC4096947

[hbm70253-bib-0014] Egerton, A. , A. Murphy , J. Donocik , et al. 2021. “Dopamine and Glutamate in Antipsychotic‐Responsive Compared With Antipsychotic‐Nonresponsive Psychosis: A Multicenter Positron Emission Tomography and Magnetic Resonance Spectroscopy Study (STRATA).” Schizophrenia Bulletin 47, no. 2: 505–516. 10.1093/schbul/sbaa128. 32910150 PMC7965076

[hbm70253-bib-0015] Ernst, M. , A. J. Zametkin , J. A. Matochik , P. H. Jons , and R. M. Cohen . 1998. “DOPA Decarboxylase Activity in Attention Deficit Hyperactivity Disorder Adults. A [Fluorine‐18]Fluorodopa Positron Emission Tomographic Study.” Journal of Neuroscience 18, no. 15: 5901–5907. 10.1523/JNEUROSCI.18-15-05901.1998. 9671677 PMC6793062

[hbm70253-bib-0016] Fornito, A. , and E. T. Bullmore . 2015. “Connectomics: A New Paradigm for Understanding Brain Disease.” European Neuropsychopharmacology 25, no. 5: 733–748. 10.1016/j.euroneuro.2014.02.011. 24726580

[hbm70253-bib-0017] Gentili, C. , L. Cecchetti , G. Handjaras , G. Lettieri , and I. A. Cristea . 2021. “The Case for Preregistering all Region of Interest (ROI) Analyses in Neuroimaging Research.” European Journal of Neuroscience 53, no. 2: 357–361. 10.1111/ejn.14954. 32852863

[hbm70253-bib-0018] Glasofer, D. R. , A. J. Brown , and M. Riegel . 2015. “Structured Clinical Interview for DSM‐IV (SCID).” In Encyclopedia of Feeding and Eating Disorders, 1–4. Springer.

[hbm70253-bib-0019] Goetz, C. G. , B. C. Tilley , S. R. Shaftman , et al. 2008. “Movement Disorder Society‐Sponsored Revision of the Unified Parkinson's Disease Rating Scale (MDS‐UPDRS): Scale Presentation and Clinimetric Testing Results.” Movement Disorders 23, no. 15: 2129–2170. 10.1002/mds.22340. 19025984

[hbm70253-bib-0020] Gunn, R. N. , S. R. Gunn , and V. J. Cunningham . 2001. “Positron Emission Tomography Compartmental Models.” Journal of Cerebral Blood Flow & Metabolism 21, no. 6: 635–652. 10.1097/00004647-200106000-00002. 11488533

[hbm70253-bib-0021] Hammers, A. , R. Allom , M. J. Koepp , et al. 2003. “Three‐Dimensional Maximum Probability Atlas of the Human Brain, With Particular Reference to the Temporal Lobe.” Human Brain Mapping 19, no. 4: 224–247. 10.1002/hbm.10123. 12874777 PMC6871794

[hbm70253-bib-0022] Hoehn, M. M. , and M. D. Yahr . 1967. “Parkinsonism.” Neurology 17, no. 5: 427. 10.1212/WNL.17.5.427. 6067254

[hbm70253-bib-0023] Howes, O. D. , B. R. Bukala , and K. Beck . 2024. “Schizophrenia: From Neurochemistry to Circuits, Symptoms and Treatments.” Nature Reviews Neurology 20, no. 1: 22–35. 10.1038/s41582-023-00904-0. 38110704

[hbm70253-bib-0024] Hughes, A. J. , S. E. Daniel , L. Kilford , and A. J. Lees . 1992. “Accuracy of Clinical Diagnosis of Idiopathic Parkinson's Disease: A Clinico‐Pathological Study of 100 Cases.” Journal of Neurology, Neurosurgery, and Psychiatry 55, no. 3: 181–184. 10.1136/jnnp.55.3.181. 1564476 PMC1014720

[hbm70253-bib-0025] Jauhar, S. , M. M. Nour , M. Veronese , et al. 2017. “A Test of the Transdiagnostic Dopamine Hypothesis of Psychosis Using Positron Emission Tomographic Imaging in Bipolar Affective Disorder and Schizophrenia.” JAMA Psychiatry 74, no. 12: 1206–1213. 10.1001/jamapsychiatry.2017.2943. 29049482 PMC6059355

[hbm70253-bib-0026] Jauhar, S. , M. Veronese , M. M. Nour , et al. 2019. “Determinants of Treatment Response in First‐Episode Psychosis: An 18F‐DOPA PET Study.” Molecular Psychiatry 24, no. 10: 1502–1512. 10.1038/s41380-018-0042-4. 29679071 PMC6331038

[hbm70253-bib-0027] Karlsgodt, K. H. , D. Sun , and T. D. Cannon . 2010. “Structural and Functional Brain Abnormalities in Schizophrenia.” Current Directions in Psychological Science 19, no. 4: 226–231. 10.1177/0963721410377601. 25414548 PMC4235761

[hbm70253-bib-0028] Kumakura, Y. , and P. Cumming . 2009. “PET Studies of Cerebral Levodopa Metabolism: A Review of Clinical Findings and Modeling Approaches.” Neuroscientist 15, no. 6: 635–650. 10.1177/1073858409338217. 19793723

[hbm70253-bib-0029] Kumakura, Y. , I. Vernaleken , H. G. Buchholz , et al. 2010. “Age‐Dependent Decline of Steady State Dopamine Storage Capacity of Human Brain: An FDOPA PET Study.” Neurobiology of Aging 31, no. 3: 447–463. 10.1016/j.neurobiolaging.2008.05.005. 18541344

[hbm70253-bib-0030] Laakso, A. , H. Vilkman , J. Bergman , et al. 2002. “Sex Differences in Striatal Presynaptic Dopamine Synthesis Capacity in Healthy Subjects.” Biological Psychiatry 52, no. 7: 759–763. 10.1016/S0006-3223(02)01369-0. 12372667

[hbm70253-bib-0031] Li, W. , N. P. Lao‐Kaim , A. A. Roussakis , et al. 2018. “11C‐PE2I and 18F‐Dopa PET for Assessing Progression Rate in Parkinson's: A Longitudinal Study.” Movement Disorders 33, no. 1: 117–127. 10.1002/mds.27183. 29082547

[hbm70253-bib-0032] Liu, X. , Y. Wang , H. Ji , K. Aihara , and L. Chen . 2016. “Personalized Characterization of Diseases Using Sample‐Specific Networks.” Nucleic Acids Research 44, no. 22: e164. 10.1093/nar/gkw772. 27596597 PMC5159538

[hbm70253-bib-0033] Liu, Z. , L. Palaniyappan , X. Wu , et al. 2021. “Resolving Heterogeneity in Schizophrenia Through a Novel Systems Approach to Brain Structure: Individualized Structural Covariance Network Analysis.” Molecular Psychiatry 26, no. 12: 7719–7731. 10.1038/s41380-021-01229-4. 34316005

[hbm70253-bib-0034] Lu, W. , T. Song , J. Li , Y. Zhang , and J. Lu . 2024. “Individual‐Specific Metabolic Network Based on (18)F‐FDG PET Revealing Multi‐Level Aberrant Metabolisms in Parkinson's Disease.” Human Brain Mapping 45, no. 14: e70026. 10.1002/hbm.70026. 39300894 PMC11413412

[hbm70253-bib-0035] Marquand, A. F. , I. Rezek , J. Buitelaar , and C. F. Beckmann . 2016. “Understanding Heterogeneity in Clinical Cohorts Using Normative Models: Beyond Case‐Control Studies.” Biological Psychiatry 80, no. 7: 552–561. 10.1016/j.biopsych.2015.12.023. 26927419 PMC5023321

[hbm70253-bib-0036] Menon, V. , L. Palaniyappan , and K. Supekar . 2023. “Integrative Brain Network and Salience Models of Psychopathology and Cognitive Dysfunction in Schizophrenia.” Biological Psychiatry 94, no. 2: 108–120. 10.1016/j.biopsych.2022.09.029. 36702660

[hbm70253-bib-0037] Moore, R. Y. , A. L. Whone , and D. J. Brooks . 2008. “Extrastriatal Monoamine Neuron Function in Parkinson's Disease: An 18F‐Dopa PET Study.” Neurobiology of Disease 29, no. 3: 381–390. 10.1016/j.nbd.2007.09.004. 18226536

[hbm70253-bib-0038] Moore, R. Y. , A. L. Whone , S. McGowan , and D. J. Brooks . 2003. “Monoamine Neuron Innervation of the Normal Human Brain: An 18F‐DOPA PET Study.” Brain Research 982, no. 2: 137–145. 10.1016/S0006-8993(03)02721-5. 12915249

[hbm70253-bib-0039] Nordio, G. , R. Easmin , A. Giacomel , et al. 2023. “An Automatic Analysis Framework for FDOPA PET Neuroimaging.” Journal of Cerebral Blood Flow & Metabolism 43, no. 8: 1285–1300. 10.1177/0271678X231168687. 37026455 PMC10369152

[hbm70253-bib-0040] Rice, J. A. 2007. Mathematical Statistics and Data Analysis. Brooks/Cole, Cengage Learning.

[hbm70253-bib-0041] Rogdaki, M. , C. Devroye , M. Ciampoli , et al. 2023. “Striatal Dopaminergic Alterations in Individuals With Copy Number Variants at the 22q11.2 Genetic Locus and Their Implications for Psychosis Risk: A [18F]‐DOPA PET Study.” Molecular Psychiatry 28, no. 5: 1995–2006. 10.1038/s41380-021-01108-y. 33981004 PMC10575769

[hbm70253-bib-0042] Rogeau, A. , A. J. Boer , E. Guedj , et al. 2025. “EANM Perspective on Clinical PET and SPECT Imaging in Schizophrenia‐Spectrum Disorders: A Systematic Review of Longitudinal Studies.” European Journal of Nuclear Medicine and Molecular Imaging 52, no. 3: 876–899. 10.1007/s00259-024-06987-1. 39576337 PMC11754335

[hbm70253-bib-0043] Sala, A. , A. Lizarraga , S. P. Caminiti , et al. 2023. “Brain Connectomics: Time for a Molecular Imaging Perspective?” Trends in Cognitive Sciences 27, no. 4: 353–366. 10.1016/j.tics.2022.11.015. 36621368 PMC10432882

[hbm70253-bib-0044] Scarpazza, C. , S. Tognin , S. Frisciata , G. Sartori , and A. Mechelli . 2015. “False Positive Rates in Voxel‐Based Morphometry Studies of the Human Brain: Should We Be Worried?” Neuroscience and Biobehavioral Reviews 52: 49–55. 10.1016/j.neubiorev.2015.02.008. 25701614

[hbm70253-bib-0045] Sedvall, G. , and L. Farde . 1995. “Chemical Brain Anatomy in Schizophrenia.” Lancet 346, no. 8977: 743–749. 10.1016/S0140-6736(95)91508-7. 7658878

[hbm70253-bib-0046] Seeley, W. W. , R. K. Crawford , J. Zhou , B. L. Miller , and M. D. Greicius . 2009. “Neurodegenerative Diseases Target Large‐Scale Human Brain Networks.” Neuron 62, no. 1: 42–52. 10.1016/j.neuron.2009.03.024. 19376066 PMC2691647

[hbm70253-bib-0047] Severino, M. , D. E. Peretti , M. Bardiau , et al. 2025. “Molecular Connectivity Studies in Neurotransmission: A Scoping Review.” Imaging Neuroscience 3. 10.1162/imag_a_00530. PMC12319898 40800788

[hbm70253-bib-0048] Sheehan, D. V. 1998. “The Mini‐International Neuropsychiatric Interview (M.I.N.I.): The Development and Validation of a Structured Diagnostic Psychiatric Interview for DSM‐IV and ICD‐10.” Journal of Clinical Psychiatry 59, no. Suppl 20: 22–33. 9881538

[hbm70253-bib-0049] Taylor, D. , C. Paton , and S. Kapur . 2012. “The Maudsley the South London and Maudsley, 11th Edition.” In NHS Foundation Trust Prescribing Guidelines in Psychiatry Eleventh Edition: The Maudsley Prescribing Guidelines in Psychiatry. NHS Foundation Trust Oxleas.

[hbm70253-bib-0050] 1993. The ICD‐10 Classification of Mental and Behavioural Disorders. Tartu Ülikool. 10.1007/BF00788743 8284737

[hbm70253-bib-0051] Tohid, H. , M. Faizan , and U. Faizan . 2015. “Alterations of the Occipital Lobe in Schizophrenia.” Neurosciences 20, no. 3: 213–224. 10.17712/nsj.2015.3.20140757. 26166588 PMC4710336

[hbm70253-bib-0052] van Erp, T. G. M. , E. Walton , D. P. Hibar , et al. 2018. “Cortical Brain Abnormalities in 4474 Individuals With Schizophrenia and 5098 Control Subjects via the Enhancing Neuro Imaging Genetics Through Meta Analysis (ENIGMA) Consortium.” Biological Psychiatry 84, no. 9: 644–654. 10.1016/j.biopsych.2018.04.023. 29960671 PMC6177304

[hbm70253-bib-0053] Verger, A. , T. Horowitz , M. B. Chawki , et al. 2020. “From Metabolic Connectivity to Molecular Connectivity: Application to Dopaminergic Pathways.” European Journal of Nuclear Medicine and Molecular Imaging 47, no. 2: 413–424. 10.1007/s00259-019-04574-3. 31741020

[hbm70253-bib-0054] Veronese, M. , L. Moro , M. Arcolin , et al. 2019. “Covariance Statistics and Network Analysis of Brain PET Imaging Studies.” Scientific Reports 9, no. 1: 2496. 10.1038/s41598-019-39005-8. 30792460 PMC6385265

[hbm70253-bib-0055] Veronese, M. , B. Santangelo , S. Jauhar , et al. 2021. “A Potential Biomarker for Treatment Stratification in Psychosis: Evaluation of an [18F] FDOPA PET Imaging Approach.” Neuropsychopharmacology 46, no. 6: 1122–1132. 10.1038/s41386-020-00866-7. 32961543 PMC8115068

[hbm70253-bib-0056] Wylie, K. P. , and J. R. Tregellas . 2010. “The Role of the Insula in Schizophrenia.” Schizophrenia Research 123, no. 2–3: 93–104. 10.1016/j.schres.2010.08.027. 20832997 PMC2957503

[hbm70253-bib-0057] Yakushev, I. , A. Drzezga , and C. Habeck . 2017. “Metabolic Connectivity: Methods and Applications.” Current Opinion in Neurology 30, no. 6: 677–685. 10.1097/WCO.0000000000000494. 28914733

[hbm70253-bib-0058] Youdim, M. B. H. , D. Edmondson , and K. F. Tipton . 2006. “The Therapeutic Potential of Monoamine Oxidase Inhibitors.” Nature Reviews Neuroscience 7, no. 4: 295–309. 10.1038/nrn1883. 16552415

[hbm70253-bib-0059] Yung, A. R. , A. R. Yung , H. Pan Yuen , et al. 2005. “Mapping the Onset of Psychosis: The Comprehensive Assessment of at‐Risk Mental States.” Australian and New Zealand Journal of Psychiatry 39, no. 11–12: 964–971. 10.1080/j.1440-1614.2005.01714.x. 16343296

